# The Clinical Pharmacokinetics and Pharmacodynamics of Warfarin When Combined with Compound Danshen: A Case Study for Combined Treatment of Coronary Heart Diseases with Atrial Fibrillation

**DOI:** 10.3389/fphar.2017.00826

**Published:** 2017-11-21

**Authors:** Chunxiao Lv, Changxiao Liu, Zhuhua Yao, Xiumei Gao, Lanjun Sun, Jia Liu, Haibo Song, Ziqiang Li, Xi Du, Jinxia Sun, Yanfen Li, Kui Ye, Ruihua Wang, Yuhong Huang

**Affiliations:** ^1^Department of Clinical Pharmacology, Second Affiliated Hospital of Tianjin University of Traditional Chinese Medicine, Tianjin, China; ^2^State Key Laboratory of Drug Delivery Technology and Pharmacokinetics, Tianjin Institute of Pharmaceutical Research, Tianjin, China; ^3^Department of Cardiology, People’s Hospital of Tianjin, Tianjin, China; ^4^Engineering Research Center of Modern Chinese Medicine Discovery and Preparation Technique, Tianjin University of Traditional Chinese Medicine, Tianjin, China; ^5^Department of Cardiology, Second Affiliated Hospital of Tianjin University of Traditional Chinese Medicine, Tianjin, China; ^6^National Center for ADR Monitoring of China, Center for Drug Reevaluation of CFDA, Beijing, China; ^7^Department of Vascular Surgery, Tianjin 4th Center Hospital, Tianjin, China

**Keywords:** warfarin, compound Danshen dripping pill, pharmacokinetics, pharmacodynamics, patients, coronary heart diseases, atrial fibrillation

## Abstract

Warfarin is used as anticoagulant and Compound Danshen prescription (CDP) is able to promote blood circulation. The combination might produce a synergic effect for patients of coronary heart diseases (CHDs) with atrial fibrillation (AF). Whether the combination increases the bleeding risk of warfarin is unclear, so the effects of Compound Danshen dripping pill (CDDP) on the pharmacokinetics (PK) and pharmacodynamics (PD) profiles of warfarin was investigated in patients. The dose and blood concentrations of warfarin, the four indicators of blood coagulation, prothrombin time, activated partial thromboplatin time, thrombin time, fibrinogen, and international normalized ratio value were compared when with and without CDDP treatment. The population PK (PPK) and PPK-PD models were established to assess patient demographics, genetic polymorphisms and CDDP as covariates. And the Seattle Angina Questionnaire was used to evaluate clinical efficacy, and the bleeding risk of combination was analyzed. The results indicated that CDDP had little influence on PK and PD profiles of warfarin in most patients and the combination of CCDP and warfarin would be a promising alternative regime for CHD with AF patients. The study was registered on China Clinical Trial Registry with number ChiCTR-ONRC-13003523.

## Introduction

It was reported that about 10%∼15% patients in coronary heart diseases (CHDs) would be associated with atrial fibrillation (AF). Moreover, about 30% patients in AF would be associated with CHD (Jason et al., 2014). The main hazard of AF is thromboembolic complications. Anticoagulants may reduce the risk of death rate in AF patients by 38% (Alpesh, 2013), so about 70–80% patients with AF are suitable for long-term use of warfarin, which was initially used in humans in the early 1950s as vitamin K antagonist (Liu et al., 2014; Darcy et al., 2017).

It is more than 100 years history in China that Compound Danshen prescription (CDP) has being applied to treat CHD, which consists of *Radix salvia miltiorrhizae, Radix notoginseng*, and *Borneolum* (Xin et al., 2013). In order to get market approval in the United States, the Compound Danshen dripping pill (CDDP), one Chinese patent drug of CDP, had recently completed a multinational phase III clinical trial. Pre-clinical and clinical studies have suggested that CDDP may increase coronary flow-rate and superoxide dismutase activity, expand blood vessel, promote blood circulation, relieve blood stasis, improve microcirculation, and improve hemorheological property, as well as decrease myocardial oxygen consumption (Pei et al., 2004; Zheng et al., 2007).

Concurrent use of CDDP with warfarin may be a desirable combination that may produce a synergistic effect, to relieve the symptoms of CHD with AF by CDDP part and meanwhile to decrease the incidence of thromboembolic complication by warfarin. Warfarin treatment is difficult to handle due to its narrow therapeutic window with a large inter-individual variability in the dose-response relationship (Zeng et al., 2016). Both pharmacodynamic (PD) and pharmacokinetic (PK) factors may contribute to more than 10–20 fold inter-individual variability in dose requirement (Hamberg et al., 2007; Steven et al., 2011; Zeng et al., 2016). Whether the CDDP could have impact on the PK and/or PD characteristics of warfarin increasing the bleeding risk as a result, is not clear. It is necessary, therefore, to get the information about the interactions between CDDP and warfarin. Only one literature report has been retrieved to address the interactions between CDDP and warfarin in rats (Chu et al., 2011), but any information on such interactions in humans has not been reported.

We conducted the study to explore the potential effects of CDDP on the PK and PD of warfarin in patients. During two periods on and off CDDP, we collected the dose and blood concentration of warfarin, the four indicators of blood coagulation, international normalized ratio (INR) value, and to establish appropriate population pharmacokinetics (PPK) and population pharmacodynamics (PPD) models to assess patient demographics, genetic polymorphisms and CDDP as covariates to evaluate the interaction effects of CDDP on warfarin. The seattle angina questionnaire (SAQ) was used to evaluate the effect of warfarin combined with CDDP on CHD with AF patients. In addition, 2 years follow-up was done after the two periods to learn about the drug compliance, the incidence of bleeding and other important outcomes, such as myocardial infarction, severe arrhythmia, revascularization, death and so on. We hope the study could provide useful clinical information for patients of CHD with AF.

## Materials and Methods

### Patients

The study was conducted in four hospitals in Tianjin from November 2013 to January 2016. Participants, suffered from CHD with AF, had been administrated warfarin with a long time. The study included two periods, in the first period patients took warfarin (Orion Corporation, Finland) at dose titration manner guided by INR values on the daily determined basis to guarantee the INR value maintained in the range of 2–3. When the INR value reached stable and maintained for successive 2 weeks on the fixed dose of warfarin, then the participants switched to the second period, in which, 10 dripping pills of CDDP (Tianjin Tasly Group Co., Ltd, China) were added orally to patients three times per day for at least 4 weeks. The dose of warfarin was re-adjusted according to the changed INR value, until the INR value was stable again and the dose of warfarin was retained for 2 weeks. Four blood samples (4^∗^3 ml) were collected at the end of each period for warfarin concentration assay. The sampling time points were arranged at trough (before the administration of warfarin), at peak, two random times on elimination phase (before the next dose of warfarin). All the time points of blood sampling and warfarin dosing were recorded accurately.

This study was carried out in accordance with the recommendations of Ethics committees of the Second Affiliated Hospital of Tianjin University of TCM with written informed consent from (ICF) all subjects. All subjects gave written informed consent in accordance with the Declaration of Helsinki. The protocol was approved by the Ethics committees of the Second Affiliated Hospital of Tianjin University of TCM in August 2013.

### Genotyping

The information of genes which affect the metabolism of warfarin was obtained by literatures (Lin G.G. et al., 2015; Zeng et al., 2016). In this study, genotyping of *VKORC1, CYP2C9^∗^3, CYP4F2, EPHX1*, and *PROC* were detected. Genomic DNA was isolated from peripheral blood leukocytes using a Genomic DNA Purification kit. Individual single-nucleotide polymorphism (SNP) loci were amplified using the polymerase chain reaction, which provided a template for allele-specific primer extension. All genotyping were performed using the gene sequencing methods (Kumar et al., 2008; Jorgensen et al., 2009; Steven et al., 2011; Lin G.G. et al., 2015). The *VKORC1* was classified by detection of 1173 C > T variant (rs9923231), the *CYP2C9^∗^3* was classified by detection of 1075 A > C variant (rs1057910), the *CYP4F2* was classified by detection of C > T variant (rs2108622), the *EPHX1* was classified by detection of G > A variant (rs2292566), the *PROC* was classified by detection of G > T variant (rs5936).

### Bioanalysis

Plasma warfarin concentrations were determined using a high-performance liquid chromatography tandem mass spectrometry method. Good chromatographic separation was achieved on an Astec CHIROBIOTIC V column (250 mm × 4.6 mm i.d., particle size 5 μm) with acetonitrile-5 mm ammonium acetate with 0.1% acetic acid in water (30:70, v/v) as the mobile phase at a flow rate of 0.50 mL/min (Jin et al., 2012). The column effluent was analyzed using a mass spectrometer in multiple reactions monitoring (MRM) mode by AB Triple Quad 5500 system in positive mode. S-warfarin, R-warfarin, and Tolglybutamide (Internal standard, IS) were extracted from plasma samples by protein precipitation with acetonitrile. Calibration curves were linear with 50.00–2000 ng/ml for S-warfarin and R-warfarin. Both intra-day and inter-day precision and accuracy of S-warfarin and R-warfarin were well within acceptance criteria (15%). The mean absolute extraction recoveries of S-warfarin, R-warfarin, and IS from human plasma were all more than 60.00%. The validated method has been successfully applied to determine of S-warfarin and R-warfarin in human plasma. Then total warfarin was the sum of S-warfarin and R-warfarin.

### Software

The population PK and PK-PD models were developed using a nonlinear mixed-effect modeling approached with the NONMEM^TM^ (nonlinear mixed-effect modeling, version VII, level 2.0, ICON Development Solutions, Ellicott City, MD, United States). Goodness-of-fit diagnostic plots were prepared with R software (3.2.1, R-project. org). All models were run using the first-order conditional estimation method with interaction (FOCEI).

### PK Model Development

#### Structural Model

After inspection of the PK profiles, a one-compartment model with first-order absorption was adopted as the optimal base model for warfarin, R-warfarin, and S-warfarin. Structural PK model was fit to plasma concentrations, and typical values of absorption rate constant (Ka), apparent volume of distribution (V/F), and oral clearance (CL/F) were calculated (where *F* denotes bioavailability). In this study, each individual parameter was expressed approximately as a coefficient of variation to be a log-normal distribution with the mean of population parameters according to results of previous researches (Maria et al., 2003; Eunice et al., 2010; Mark et al., 2010; Juno et al., 2013).

(1) $$\begin{array}{c} P_{ij}=P_{TV\;j}\cdot Exp(\eta_{ij}) \end{array}$$

Where P_ij_ was the PK Parameters *j* for *ith* individual, P_TV j_ was mean of predicted population of PK Parameters *j*, η_ij_ was a between-subject random variable distributed normally. PK Parameters *j* was just about Ka, V/F, or CL/F.

The residual error model was assumed to be a mixed error model as following:

(2) $$\begin{array}{c} C_{Obs}=C_{Pred}\cdot (1+\varepsilon_{1})+\;\varepsilon_{2} \end{array}$$

Where C_Obs_ was the observed plasma concentration, C_Pred_ was the model prediction concentration. Both of multiplicative residual error (𝜀_1_) and additive residual error (𝜀_2_) were assumed as a normal distribution.

#### Covariate Models

These covariates were first explored graphically and each potential covariate individually added to the base model if graphical trends were shown. For the covariate models, stepwise of a forward inclusion step and a backward elimination step method was used. When a variable was considered for entering in the final model, it must reduce the objective function value (OFV) by more than 3.84 if *p* < 0.05 (5% significance level assuming a one degree of freedom, ΔOFV > 3.84, df = 1; ΔOFV > 5.99, df = 2; ΔOFV > 7.81, df = 3). The variable that had the biggest impact on the OFV could enter first and subsequent variables added according to their impact on the OFV. The forward process described above was repeated again until no further covariates were incorporated into the model. Then, the backward elimination step was implementing. The variables were retained in the model if its removal caused an increase in OFV at least 6.63 if *p* < 0.05(ΔOFV > 6.63, df = 1). The relative contribution of each covariate to the goodness of fit was evaluated by deleting it from the full model. With these restrictive criteria, only covariates showing statistically significant and clinically relevant contributions were kept in the population PK (PPK) model.

In our research, the covariate of weight was described by allometric scaling equation:

(3) $$\begin{array}{c} P_{i,j}=P_{TV}\cdot {\left(\frac{WT}{\bar{WT}}\right)}^{\theta_{2}}\cdot Exp(\eta_{i,j}) \end{array}$$

Where *P_i,j_* was the PPK parameters, *P_TV_* was a reference value of PPK parameters, *WT* was weight, $\bar{WT}$ was median of *WT*,𝜃_2_ was effect value of *WT* to PPK parameter.

Other covariates (except weight) were divided into categorical covariates (gender, genotyping, administrated CDDP) and continuous covariates (age, BMI, ALT, AST, BUN, CRE, CRCL).

For the categorical covariates, the following equation was adopted:

(4) $$\begin{array}{c} If(COV=l)P_{i,j}=(P_{TV}+\theta_{l})\cdot Exp(\eta_{i,j})l=2,...m \end{array}$$

Where *COV* was a categorical covariate which had *m* levels, 𝜃_l_ was adjusted value for *P_TV_* to *P_i,j_.*

For categorical covariates, the linear models were employed:

(5) $$\begin{array}{c} P_{i,j}=P_{TV}\cdot (1+(COV-\bar{COV})\cdot \;\theta_{3})\cdot Exp(\eta_{i,j})\;\left(5\right) \end{array}$$

Where COV was continuous covariates, $\bar{COV}$ was median of continuous covariate, 𝜃_3_ was a coefficient for the effect of covariate to parameter.

### PK–PD Model Development

The PK–PD model was developed only for S-warfarin, because S-warfarin is the main active ingredient, which is 3–5 times more potent than R-warfarin. According to the plot of relationship between INR and concentration of S-warfarin, the Emax model was selected as the PD model. For the covariate models, stepwise of forward and backward method was used as mentioned in PK model.

### Model Evaluation

#### Model Diagnostics

The PK models for warfarin, R-warfarin, S-warfarin, and PK-PD model for S-warfarin was evaluated by the goodness of fit of these models using visual inspection of diagnostic scatter plots of the observed plasma concentrations (DV) versus mean population predicted plasma concentrations (PRED), DV versus individual predicted plasma concentrations (IPRED), conditional weighted residuals (CWRES) versus time, individual weighted residuals (IWRES) versus population predictions (PRED).

#### Model Validation

##### Visual predictive check (VPC)

A visual predictive check (VPC) was performed to evaluate the prediction of PK models for warfarin, R-warfarin, S-warfarin, and PK-PD model for S-warfarin. The VPC were conducted by comparing 1000 datasets simulated from the final parameters with the observed plasma concentrations. The 95% predicted intervals (PIs) obtained from the simulation were superimposed and compared with the observations.

##### Bootstrap

A nonparametric bootstrap analysis was used to assess the stability of the parameter estimates and to confirm the robustness of the models. The 1000 bootstrap sample datasets were re-sampled from random sampling with replacement from the original data using individual as sampling unit. Next, population parameters of final PK and PK-PD models for each dataset were estimated. Then, the median and 95% confidence intervals (CI) were constructed by obtaining the 2.5th and 97.5th smallest values out of 1000 parameters estimated from bootstrap sample datasets. Comparing with the mean and 95% CI, each estimated parameter derived from the mean and its standard error of the final parameters.

### SAQ and Follow-Up

The SAQ was applied to evaluate clinical symptoms of patients. The score of SAQ was collected after each period of clinical trial, and compared by paired *t*-test. In order to learn about more information about warfarin and CDDP administration, the follow-ups were done every 6 months and lasted for 2 years. The contents of follow-up included the length of taking the combination of warfarin and CDDP and some important and main outcomes, such as bleeding, myocardial infarction, severe arrhythmia, revascularization, ACS, TIA, stroke, heart failure, death, and other conditions causing hospitalization.

## Results

### Patients

**FIGURE 1:**
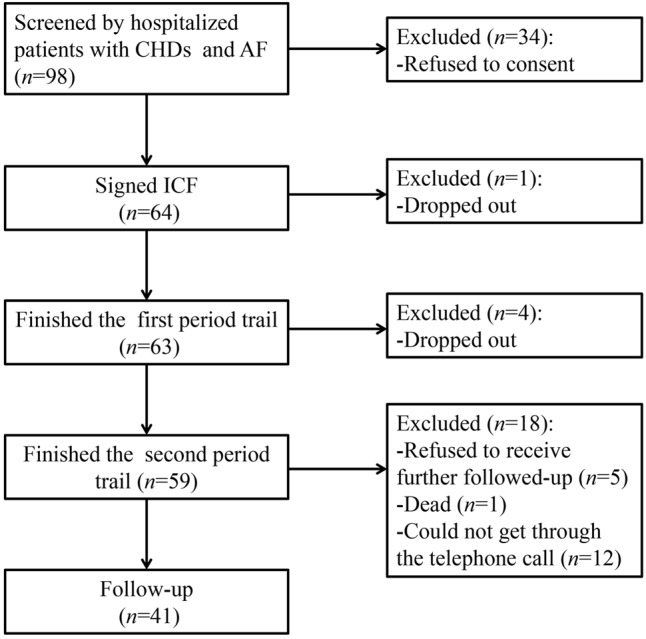
The participant flow chart.

Sixty-four patients were enrolled from four hospitals in Tianjin from November 2013 to January 2016. Fifty-nine patients completed the trial, among which 21 patients were female, and the mean age was 63 years (49–79 years). The demographic information of the patients was presented in **Table 1**. There were 404 samples and 600 INR values available for analysis from the 59 patients. About 41 patients completed the follow-up. The participant flow chart was shown in **Figure 1**.

**Table 1:** The demographic profile summary for subjects.

Characteristics	Values (Range)
Number of patients	59
Number of plasma concentration points	404
Number of international normalized ratio (INR)	600
Dose of warfarin (mg)	3.41 (0.75-7.50)
Concentration of S-warfarin (mg/L)	0.43 (0.10-1.74)
Concentration of R-warfarin (mg/L)	0.89 (0.19-3.11)
Concentration of Total-warfarin (mg/L)	1.32 (0.29-4.85)
INR	2.17 (0.89-4.83)
Gender (male/female)	38/21
Age (year)	63 (49-79)
Weight (WT, Kg)	73 (50-99)
BMI (kg/m^2^)	25.70 (16.03-34.48)
*VKORC1* (%)	
T/T	54 (91.53%)
C/T	5 (8.47%)
*CYP2C9^∗^3* (%)	
A/A	51 (86.44%)
A/C	8 (13.56%)
*CYP4F2* (%)	
C/C	35 (59.32%)
C/T	19 (32.20%)
T/T	5 (8.47%)
*EPHX1* (%)	
A/A	2 (3.39%)
A/G	33 (55.93%)
G/G	24 (40.68%)
*PROC* (%)	
G/G	11 (18.64%)
T/T	13 (22.03%)
T/G	35 (59.32%)
Alanine aminotransferase, ALT (U/L)	28.7 (10-137)
Aspartate aminotransferase, AST (U/L)	22.9 (8-80)
Blood urea nitrogen, BUN (mmol/L)	5.45 (2.30-13.70)
Creatinine, CRE (mmol/L)	81.64 (48-278)
Creatinine clearance rate, CRCL (ml/min)	85.39 (19.15-158.7)
Systolic blood pressure, SBP (mmhg)	126.73 (93-180)
Diastolic blood pressure, DBP (mmhg)	78.09 (60-105)
Long use (LU) of warfarin	32 (54.24%)

### Fixed Dose and Concentration of Warfarin

In the two periods, the fixed doses of warfarin were 3.39 ± 1.04 mg and 3.36 ± 0.92 mg (*P* = 0.691), respectively. The steady-state concentration in the two periods was 1.2225 ± 0.5329 mg kg^-1^ and 1.1849 ± 0.4949 mg kg^-1^ (*P* = 0.587) for warfarin, 0.8337 ± 0.3597 mg kg^-1^ and 0.8128 ± 0.3132 mg kg^-1^ (*P* = 0.650) for R- warfarin, 0.3887 ± 0.2253 mg kg^-1^ and 0.3721 ± 0.2301 mg kg^-1^ (*P* = 0.535) for S-warfarin. So, the fixed dose and the steady-state concentration of warfarin were no statistically different between warfarin alone and warfarin plus CDDP. The results were shown in **Table 2**.

**Table 2:** The fixed dose and blood concentrations for patients in the first and second period.

	Mean	*SD*	*P*
**Fixed doe of warfarin (mg)**			
First period	3.39	1.04	0.691
Second period	3.36	0.92	
**Blood concentrations of warfarin (mg/L)**			
First period (warfarin)	1.2225	0.5329	0.587
Second period (warfarin)	1.1849	0.4949	
First period (R-warfarin)	0.8337	0.3597	0.650
Second period (R-warfarin)	0.8128	0.3132	
First period (S-warfarin)	0.3887	0.2253	0.535
Second period (S-warfarin)	0.3721	0.2301	

### Four Indicators of Blood Coagulation and INR Value

The results were shown in **Table 3**. The four indicators of blood coagulation, prothrombin time (PT), activated partial thromboplatin time (APTT), thrombin time (TT), fibrinogen (FIB), and INR value between the two periods at the fixed warfarin dose had no statistical differences which was compared by paired *t*-test.

**Table 3:** The INR value, four indicators of blood coagulation, and seattle angina questionnaire (SAQ) for patients in the first and second period.

Mean ± SD	INR value	PT (s)	APTT (s)	TT (s)	FIB (g/L)	SAQ (Score)
First period	2.42 ± 0.29	25.92 ± 2.43	47.74 ± 10.48	16.95 ± 1.64	3.47 ± 0.55	19.71 ± 5.05
Second period	2.40 ± 0.61	25.64 ± 5.01	49.24 ± 9.03	17.49 ± 1.60	3.49 ± 0.80	21.02 ± 5.07
*P*	0.893	0.803	0.514	0.120	0.874	0.002

### Structural Model for PK Models

The PK profile of warfarin was in accordance with one-compartment model (Eunice et al., 2010; Steven et al., 2011) or two-compartment model (Jiang et al., 2006; Hamberg et al., 2007) based on literatures, including warfarin, S-warfarin, and R-warfarin. One-compartment and two-compartment models were both investigated for warfarin, S-warfarin and R-warfarin in this study. By comparing OFVs, goodness of fit to the models, as well as rational of parameters, one-compartment was chosen as the optimal ones for initial structure models for warfarin, S-warfarin, and R-warfarin.

### Covariate Models for PK Models

Once the base structural models were established, potentially significant covariates were evaluated as described. The OFVs of structure models for warfarin, R-warfarin, and S-warfarin were –622.414, –897.240, and –1448.94. When the covariate of weight was on CL, OFV were –623.958, –898.783, and –1449.40 for warfarin, R-warfarin, and S-warfarin, respectively. When the covariate of weight was on V, OFV were –622.423, –897.335, and –1448.94 for warfarin, R-warfarin and S-warfarin, respectively. Comparing with structural models, these OFVs were not more than 3.84, so weight was not the significantly covariates for warfarin, R-warfarin, and S-warfarin.

For warfarin models, in the forward models, three covariates were brought in, *PROC* on CL, LU on CL, *PROC* on Ka, but in the backward models, LU on CL was eliminated. At last, the covariates of *PROC* on Cl and *PROC* on Ka were in the model. For R-warfarin models, *PROC* on Ka and *PROC* on CL were in the models, then *PROC* on CL was removed, so *PROC* on Ka was the significantly covariate. For S-warfarin model, the covariates *PROC* on Ka, CDDP on KA, *CYP2C9^∗^3* on CL, *EPHX1* on V and *VKORC1* on CL were in the forward models, then two covariates were rejected, *PROC* on Ka, CDDP on KA and *CYP2C9^∗^3* on CL were in the models as the significantly covariates. The detail information about the covariates for warfarin, R-warfarin, and S-warfarin were shown in **Table 4**.

**Table 4:** The covariates of pharmacokinetics (PK) models for Warfarin, R-warfarin, and S-warfarin.

Warfarin			R-warfarin			S-warfarin		
Description	Estimate	RSE (%)	Description	Estimate	RSE (%)	Description	Estimate	RSE (%)
Ka	0.999	43.7	Ka	0.586	129.4	Ka	1.69	74.6
CL/F	0.117	8	CL/F	0.158	3.7	CL/F	0.357	4.6
V/F	11.3	16	V/F	17.8	17.9	V/F	25.5	15.1
*PROC* on Ka(G/G)	1		*PROC* on Ka(G/G)	1		*PROC* on Ka(G/G)	1	
*PROC* on Ka(T/T)	0.0355	45.6	*PROC* on Ka(T/T)	0.055	120.9	*PROC* on Ka(T/T)	0.21	98.1
*PROC* on Ka(T/G)	4.29	60.1	*PROC* on Ka(T/G)	4.52	121.2	*PROC* on Ka(T/G)	15.7	67.5
*PROC* on CL(G/G)	1					CDDP^∗^ on Ka	-0.796	13.9
*PROC* on CL(T/T)	0.764	9.4				*CYP2C9^∗^3* on CL(A/A)	1	
*PROC* on CL(T/G)	0.941	9.1				*CYP2C9^∗^3* on CL(A/C)	0.686	14.2
IIVKa/F	0(FIX)		IIVKa/F	0.206	115.3	IIVKa/F	2.29	39.1
IIV CL/F	0.0552	8.8	IIV CL/F	0.072	9.4	IIV CL/F	0.105	7
IIV V/F	0.501	17	IIV V/F	0.588	18.9	IIV V/F	0.438	20
Prop.RE (sd)	0.172	8.8	Prop.RE (sd)	0.182	10.9	Prop.RE (sd)	0.18	7.3
Add.RE (sd)	0(FIX)		Add.RE (sd)	0(FIX)		Add.RE (sd)	0(FIX)	

*^∗^When CDDP was 0 represented the first period that patients was not administrated CDDP, and CDDP was 1 represented the second period that patients was administrated CDDP.*

### PK–PD Model

On the basis of the final PK model, individual estimates of S-warfarin concentrations were predicted and used in the development of PD model. Graphical analyses of the INR observations versus time for S-warfarin demonstrated the Emax model may be more suitable for PD model. So direct Emax models, as well as BIOPH Emax models were investigated in our research. The parameters of the two models were all within a reasonable range, but OFV of BIOPH Emax model decreased 16.35 compared with direct Emax model. Moreover, there was a considerable time delay between INR response and drug concentration. Therefore, BIOPH Emax model was at last considered as the PD model of S-warfarin.

Once the basic structural model was established, potentially significant covariates were evaluated. From the consequence of forward and backward method and the reasonable parameters, AST was finally brought on KE0 of PD model for S-warfarin. The detail information about covariates of PK-PD model for S-warfarin was shown in **Table 5**.

**Table 5:** The covariates of pharmacokinetics–pharmacodynamics (PK–PD) models for S-warfarin.

Description	Estimate	RSE (%)
KA	1.44	44.2
CL/F	0.356	4.7
V/F	26.1	14.3
*PROC* on Ka(T/T)	0.272	64
*PROC* on Ka(T/G)	12.2	65.1
CDDP^∗^ on Ka	-0.728	20.1
*CYP2C9^∗^3* on CL(A/C)	0.69	14.5
KE0	0.0365	48.2
E0	1.21	4.9
EMAX	3.54	81.1
EC50	0.889	113.6
AST ON KE0	0.0006	0.7
IIV KA	2.14	58.9
IIV CL/F	0.0984	14.5
IIV V/F	0.503	41.6
IIVE0	0.0196	54.6
IIVEC50	0.198	31.6
Residual errors		
PK Prop.RE (*SD*)	0.014	7.7
PK Add.RE (*SD*)	0 (FIX)	
PD Prop.RE (*SD*)	0.0112	5.3
PD Add.RE (*SD*)	0 (FIX)	

*^∗^When CDDP was 0 represented the first period that patients was not administrated CDDP, and CDDP was 1 represented the second period that patients was administrated CDDP.*

### Model Evaluation

#### Model Diagnostics

**FIGURE 2:**
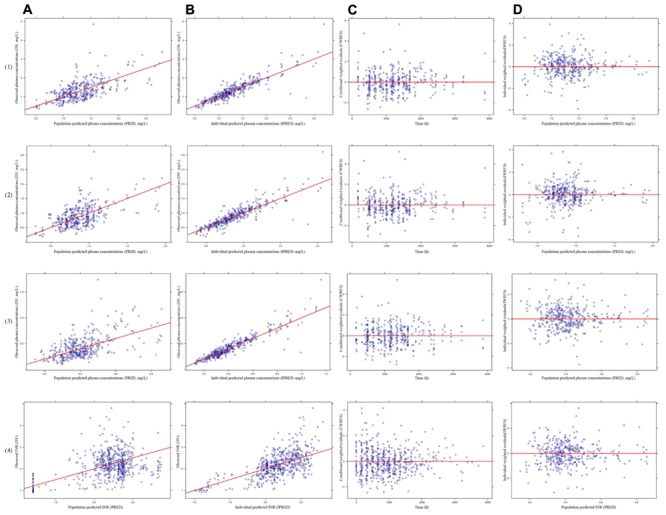
The model diagnostics plots of pharmacokinetics (PK) models for warfarin (1), R-warfarin (2) and S-warfarin (3) and pharmacokinetics–pharmacodynamics (PK–PD) model for S-warfarin (4). **(A)** The observed plasma concentrations (DV) versus mean population predicted plasma concentrations (PRED), **(B)** The DV versus individual predicted plasma concentrations (IPRED), **(C)** The conditional weighted residuals (CWRES) versus time, **(D)** The individual weighted residuals (IWRES) versus population predictions (PRED).

The model diagnostic plots were shown in **Figure 2**. **Figures 2A,B** demonstrated that all the data points distributed uniformly in both sides of line *y* = *x*. **Figures 2C,D** demonstrated that CWRES and IWRES distributed uniformly in both sides of line *y* = 0, and the absolute value were less than 4. So, these models adequately described the plasma concentrations, suggesting good fitness of the PK models for warfarin, R-warfarin, S-warfarin, and PK-PD model for S-warfarin.

#### Visual Predictive Check

**FIGURE 3:**
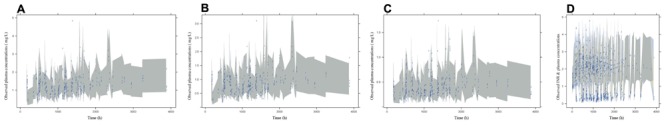
The VPC plots of PK models for warfarin **(A)**, R-warfarin **(B)**, and S-warfarin **(C)** and PK-PD model for S-warfarin **(D)**. The Shadows were the 95th PIs.

The VPC plots were shown in **Figure 3**. Most of the observations were in the 95% PIs, so the fit of the models were acceptable in terms of visual or statistical biases for the prediction.

#### Bootstrap

The estimated parameters and 95% values from all bootstrap runs for the PK models of warfarin, R-warfarin, and PK-PD model of S-warfarin were given in **Table 6**. The data indicated that the parameter estimated in PK models and PK-PD model had little bias and the models were fairly robust.

**Table 6:** The bootstrap results of PK models for warfarin, R-warfarin and PK-PD model for S-warfarin.

Warfarin					R-Warfarin					S-Warfarin				
Description	Model	Bootstrap (95%CI)			Description	Model	Bootstrap (95%CI)			Description	Model	Bootstrap (95%CI)		
	Median	Median	2.5%	97.5%		Median	Median	2.5%	97.5%		Median	Median	2.5%	97.5%
Ka	0.999	0.839	0.114	3.42	Ka	0.586	0.258	0.0695	0.965	KA	1.44	1.82	0.613	11.9
CL/F	0.117	0.116	0.094	0.145	CL/F	0.158	0.158	0.151	0.169	CL/F	0.356	0.359	0.333	0.381
V/F	11.3	10.6	7.36	14.7	V/F	17.8	15.3	10.5	23.7	V/F	26.1	24.4	18.3	33.1
*PROC* on Ka(T/T)	0.0355	0.0478	0.0102	1.12	*PROC* on Ka(T/T)	0.055	0.108	0.0224	0.400	*PROC* on Ka(T/T)	0.272	0.171	0.00849	2.93
*PROC* on Ka(T/G)	4.29	4.42	0.689	45.6	*PROC* on Ka(T/G)	4.52	8.11	1.68	59.6	*PROC* on Ka(T/G)	12.2	7.69	1.25	58.74
*PROC* on CL(T/T)	0.764	0.772	0.608	0.957						CDDP^∗^ on Ka	-0.728	-0.756	-0.866	0.095
*PROC* on CL(T/G)	0.941	0.922	0.691	1.13						*CYP2C9^∗^3* on CL(A/C)	0.69	0.65	0.52	0.95
										KE0	0.0365	0.0331	0.0254	0.0367
										E0	1.21	1.30	1.16	1.48
										EMAX	3.54	3.63	3.23	3.79
										EC50	0.889	0.921	0.723	1.31
										AST ON KE0	0.0006	0.0004	0.0001	0.0011
IIVKa/F	0(FIX)	0	0	0	IIVKa/F	0.206	0.275	0.00280	0.974	IIV KA	2.14	2.35	0.52	5.08
IIV CL/F	0.0552	0.0514	0.0307	0.0772	IIV CL/F	0.072	0.063	0.046	0.089	IIV CL/F	0.0984	0.0936	0.0724	0.118
IIV V/F	0.501	0.538	0.193	1.162	IIV V/F	0.588	0.483	0.284	0.791	IIV V/F	0.503	0.396	0.145	0.917
										IIVE0	0.0196	0.015	0.009	0.024
										IIVEC50	0.198	0.141	0.124	0.297
Prop.RE (sd)	0.172	0.168	0.134	0.201	Prop.RE (sd)	0.182	0.196	0.153	0.230	PK Prop.RE(sd)	0.014	0.017	0.015	0.020
Add.RE (sd)	0(FIX)	0	0	0	Add.RE (sd)	0(FIX)	0	0	0	PK Add.RE (sd)	0(FIX)	0	0	0
										PD Prop.RE (sd)	0.0112	0.013	0.0089	0.018
										PD Add.RE (sd)	0(FIX)	0	0	0

### The Results of SAQ and Follow-Up

The results of SAQ were shown in **Table 3**. The score of SAQ during trail were 19.71 ± 5.05 for the first period and 21.02 ± 5.07 for the second period. There was significant difference between two periods (*P* = 0.002). During 2 years of follow-up, the mean length of taking CDDP is 0.96 ± 0.80 years and 1.70 ± 0.83 years for warfarin. Severe arrhythmia occurred in one patient, revascularization in two patients, death in one patient, and 16 patients were hospitalized due to other conditions. The incidence of severe arrhythmia, revascularization, death, and hospitalization were 2.44% (1/41), 4.88% (2/41), 2.44% (1/41), and 39.02% (16/41).

## Discussion

In this study a total of 404 blood samples, instead of 472 as required by protocol (eight for each patient), were collected for warfarin assay. There were 600 INR values obtained in the study. Being serious in nature of the heart disease, the compliance of the enrolled patients in the study was relatively low. With the limited number of patients enrolled in this study, we use the principle of PPK algorithm with more detail information collected from the participants, as described in the Guidance for Industry PPK published by FDA which states “Since patients are studied in more detail in this design, the design requires fewer subjects, and the relationship of trough levels to patient characteristics can be evaluated with more precision”. Similarly, in some other PPK-PPD studies (Hamberg et al., 2007; Parker et al., 2015; Agarwal et al., 2016; Pier et al., 2017), these numbers of subjects enrolled were comparable to our study.

These CHD patients with AF had been taking warfarin for a long period of time to decrease the risk of thromboembolic complications. The underlying conditions included hypertension (28.8%), diabetes (18.7%), and cerebral ischemic stroke (16.9%) in the study. Various medications were concomitantly used in the patients due to its complicated property of the disease. More frequently used drugs included β-blockers (39.0%), nitric esters (33.9%), statins (22.0%), diuretics (22.0%), cardiac glycosides (18.7%), calcium channel blockers (15.3%), and antidiabetic drugs (15.3%). Identification of the effect of CDDP on profiles of PK and PD of warfarin were established on a self-control design, maintaining all concomitant medications except warfarin from beginning to end of trial. The INR value, fixed warfarin doses and trough concentration of plasma warfarin had not significantly difference (*P* > 0.05) with or without CDDP. That suggested there is no drug-drug interaction between CDDP and warfarin in human, and CDDP did not affect anticoagulant mechanism of warfarin because CDDP did not interfere metabolic process of warfarin in human.

The PPK models for warfarin, S-warfarin and R-warfarin and PPD model for S-warfarin were developed with assessing patient demographics, genetic polymorphisms and CDDP as covariates. The PK behavior of warfarin, S-warfarin and R-warfarin was in accordance with one-compartment model or two-compartment model based on literatures (Jiang et al., 2006; Hamberg et al., 2007; Eunice et al., 2010; Steven et al., 2011). The One-compartment models were more optimal and reasonable for warfarin, either S-warfarin or R-warfarin in our study. The consequence of *CYP2C9^∗^3* on CL of S-warfarin was identical to other researches (Chanan et al., 2016; Darcy et al., 2017), but the age, gender, weight had no significant effects on S-warfarin or R-warfarin which were inconsistent with some research (Hamberg et al., 2007; John et al., 2012; Lin R.F. et al., 2015). The gene of *CYP4F2, PROC, VKOR, CYP2C9^∗^3*, and *EPHX1* were investigated in this research. There was no precise conclusion about the effects of gene subtypes on PK and PD characteristics of warfarin due to limited distribution rate of each individual subtype, which was consistent with other literatures (Özer et al., 2011; Radka et al., 2015). The change of the fixed dose of warfarin in EPHXI gene subtype A/A is higher than the other gene types. Because only two patients with *EPHX1* gene subtype A/A were enrolled, it is difficult to make the conclusion that CDDP affect the PK and PD characteristics of warfarin on this kind of patient.

The study result also suggested by PPK model and PPD model that there may be no influence of CDDP on PK and PD of warfarin in patients, although CDDP was as a covariate on Ka of S-warfarin. There had a great variation of Ka with a higher RSE (43.7%, 129.4%, 74.6% for PK models of warfarin, R- warfarin, and S- warfarin) meaning an incredible consequence about absorption. The bioavailability of warfarin is more than 95% (Mark et al., 2010; Juno et al., 2013), some reports had applied 100% bioavailability when developing models (Maria et al., 2003; Hamberg et al., 2007; Steven et al., 2011). Moreover, there were few reports to evaluate the absorption of warfarin due to its high bioavailability.

The SAQ was applied to evaluate efficacy effect of the co-treatment of warfarin and CDDP in the patients of CHD with AF. It’s showed that there was significant difference in the SAQ score with or without CDDP. It may indicate that CDDP can improve the life quality of CHD with AF patients when both INR and dose of warfarin are stable. During 2 years follow-up, many patients still took the combination for a long time, and there were no report about bleeding. So, the combination of CDDP with warfarin might relieve clinical symptoms and provide benefits for patients with CHD and AF. However, this was not a randomized clinical trial, some researches would be needed to further demonstrate the clinical efficacy.

## Conclusion

In summary, robust and stable PK-PD models have been successfully developed for evaluating the effect of CDDP on the PK and PD of warfarin. The results indicated that CDDP did not influence the INR stability and PK characteristic of warfarin when warfarin was administrated simultaneously with CDDP in most CHDs patients. Moreover, The SAQ and follow-up results showed the CDDP combined with warfarin might provide benefit in clinical practice for patients. This study would provide some useful information of the combined regimen of CDDP and warfarin for the treatment of CHDs with AF, but the result in Chinese genetic subtypes of *EPHX1* and the clinical efficacy study need to be confirmed further.

## Author Contributions

CLv and YH wrote the manuscript. CLiu, YH, XG, and HS designed the research. ZY, LS, XD, JS, YL, and KY performed research. CLv, JL, ZL, and RW analyzed data.

## Conflict of Interest Statement

The authors declare that the research was conducted in the absence of any commercial or financial relationships that could be construed as a potential conflict of interest.
